# Differentiation of Pituitary Adenoma from Rathke Cleft Cyst: Combining MR Image Features with Texture Features

**DOI:** 10.1155/2019/6584636

**Published:** 2019-10-28

**Authors:** Yang Zhang, Chaoyue Chen, Zerong Tian, Yangfan Cheng, Jianguo Xu

**Affiliations:** ^1^Department of Neurosurgery, West China Hospital, Sichuan University, Chengdu, China; ^2^West China School of Medicine, West China Hospital, Sichuan University, Chengdu, China

## Abstract

**Objectives:**

To differentiate pituitary adenoma from Rathke cleft cyst in magnetic resonance (MR) scan by combing MR image features with texture features.

**Methods:**

A total number of 133 patients were included in this study, 83 with pituitary adenoma and 50 with Rathke cleft cyst. Qualitative MR image features and quantitative texture features were evaluated by using the chi-square tests or Mann–Whitney *U* test. Binary logistic regression analysis was conducted to investigate their ability as independent predictors. ROC analysis was conducted subsequently on the independent predictors to assess their practical value in discrimination and was used to investigate the association between two types of features.

**Results:**

Signal intensity on the contrast-enhanced image was found to be the only significantly different MR image feature between the two lesions. Two texture features from the contrast-enhanced images (Histo-Skewness and GLCM-Correlation) were found to be the independent predictors in discrimination, of which AUC values were 0.80 and 0.75, respectively. Besides, the above two texture features (Histo-Skewness and GLCM-Contrast) were suggested to be associated with signal intensity on the contrast-enhanced image.

**Conclusion:**

Signal intensity on the contrast-enhanced image was the most significant MR image feature in differentiation between pituitary adenoma and Rathke cleft cyst, and texture features also showed promising and practical ability in discrimination. Moreover, two types of features could be coordinated with each other.

## 1. Introduction

Pituitary adenoma is the most common lesion in the sellar region, accounting for 15% of all intracranial tumor [1, 2]. Rathke cleft cyst, another type of frequent lesion located in the sellar region, could be found in approximately 13%–33% of normal pituitary glands according to the routine autopsy [3, 4]. They share similar symptoms arisen from mass effect, including headache, visual deficit, and endocrine dysfunction [5, 6]. Magnetic resonance (MR) scan has long been recognized as the most useful technique to distinguish the two types of lesions, which could be discriminated based on their different features in many cases. Pituitary adenoma typically shows solid characteristics and homogeneous enhancement, while Rathke cleft cyst usually presents as a cystic lesion combined with rim enhancement [7]. However, the diagnostic accuracy of MR scan largely depends on the experiences of radiologists, and the versatile characteristics of Rathke cleft cyst make it difficult to discriminate from pituitary adenoma in some cases radiologically, requiring a more objective, precious, and practical method to depict the MR image [8, 9].

Texture analysis is a novel radiological method which could extract quantitative features from medical images and provide nonvisual information systematically and objectively through well-established mathematical formulas [10, 11]. Previous studies implied that texture analysis combined with magnetic resonance imaging (MRI) could be applied for the detection and differentiation of various tumors, such as breast, bone, bladder, and prostate tumor [12–15]. However, texture analysis has never been conducted in pituitary adenoma or Rathke cleft cyst, and the relationship between texture features and MR image features has rarely been reported in the previous study. Therefore, in the present study, we performed analysis to discriminate pituitary adenoma from Rathke cleft cyst in MR scan by combing MR image features with texture features, wishing to establish a set of parameters from macroscopical to microcosmic.

## 2. Methods

### 2.1. Patient Selection

All patients involved in this retrospective single-center study were diagnosed and treated at the neurosurgery department of the West China Hospital of Sichuan University from December 2014 to April 2019. In initial selection, we screened the database of our hospital to select the potential qualified patients who were (1) with elaborate electric medical records, (2) with the pathological confirmation of pituitary adenoma or Rathke cleft cyst, and (3) with preoperative sellar MR scan. Exclusion criteria included patients (1) with MR image which had the scarce quality to perform analysis; (2) with any other cerebral diseases which could disturb statistic extraction. A total number of 133 eligible patients were selected from our institutional database. Eighty-three of them were diagnosed with pituitary adenoma and fifty with Rathke cleft cyst.

### 2.2. MRI Acquisition

MR scans were conducted in the Huaxi MR Research Center of the West China Hospital of Sichuan University in Chengdu, Sichuan, China. Patients were scanned using the 3.0 T Siemens Trio scanner or 3.0 T GE SIGNA MRI scanner. The sequences included the contrast-enhanced image on the T1-weighted image (T1WI) and T2-weighted image (T2WI). The scanning of the contrast-enhanced image was performed within 200 s after injection of gadopentetate dimeglumine (0.1 mmol/Kg). The contrast-enhanced image on T1WI was available for all patients, while T2WI was available for 113 patients.

### 2.3. MR Image Feature Analysis

MR image feature analysis was conducted by two certificated neurosurgeons together with the assistance of senior radiologists. The two neurosurgeons correlated MRI with relevant histopathologic findings in qualitative and quantitative assessment. They were guided by the advice from senior radiologists when their opinions divided to reach an agreement. Based on the previous studies and empirical suggestions from radiologists, the qualitative MR features we selected for further analysis were as follows: (1) signal intensity of the whole lesion on the contrast-enhanced image and T2WI, (2) heterogeneity of the whole lesion on the contrast-enhanced image and T2WI, and (3) flow voids on the contrast-enhanced image or T2WI [7, 9]. In addition, characteristics of the lesion were also measured and recorded, including the size and location of lesions, to perform further research.

### 2.4. Texture Feature Extraction

The 3D-based texture feature extraction was performed by two neurosurgeons together using mature software called LifeX package (http://www.lifexsoft.org) [16]. Concerning about ambiguity on location of lesions on conventional T1WI, we selected the contrast-enhanced image and T2WI to conduct texture analysis. Two neurosurgeons reached unanimous agreement on the location of the lesion under the guidance of senior radiologists and manually contoured the lesion slice by slice on coronal image to generate regions of interest (ROI). A total of 46 quantitative texture features were extracted from five matrices, including histogram-based matrix (Histo), grey-level co-occurrence matrix (GLCM), grey-level zone length matrix (GLZLM), neighborhood grey-level dependence matrix (NGLDM), and grey-level run length matrix (GLRLM). Based on the results from the previous studies, we focused statistical analysis on ten texture features most researchers studied: energy, entropy, kurtosis, and skewness derived from histogram-based matrix and correlation, contrast, dissimilarity, energy, entropy, and homogeneity derived from GLCM [10, 17].

### 2.5. Statistical Analysis

The continuous variables were summarized with mean value and ranges, and the categorical variables were summarized with frequencies and percentages. The patients were divided into training group (*n* = 108) and validation group (*n* = 25). We first conducted MR image feature analysis and texture feature analysis in the training group, including 68 patients with pituitary adenoma and 40 patients with Rathke cleft cyst. The differences of MR image features between pituitary adenoma and Rathke cleft cyst were evaluated with the chi-squared tests (for categorical variables with enough statistics) or Fisher exact tests (for categorical variables with limited statistics). The Mann–Whitney *U* test was conducted to identify whether texture features were statistically different between two types of lesions. Binary logistic regression analysis was performed on significant features to evaluate if they could be taken as independent predictors. Receiver operating characteristic (ROC) analysis was performed on these independent predictors to assess their ability to differentiate pituitary adenoma from Rathke cleft cyst, from where area under the curve (AUC), sensitivity, specificity, and optimal cutoff values (at the maximal Youden's index) were recorded to evaluate their practical value. Then, we tested the discriminative performance of the independent texture features in the validation group, including 15 patients with pituitary adenoma and 10 patients with Rathke cleft cyst. As for the association between MR image features and texture features, the Mann–Whitney *U* test was performed first to explore whether texture features were significantly different in specific MR image features. ROC analysis was performed subsequently to investigate their relationship and practical value. Benjamini–Hochberg correction was applied for all multiple hypothesis tests in order to control the true type-1 error probability [18, 19]. *P* values less than 0.05 were considered statistically significant. All statistical analyses were performed with IBM SPSS Statistics for Windows (Version 22.0, IBM Corp. Armonk, NY, USA) and MedCalc statistics (MedCalc Software, Belgium).

## 3. Results

### 3.1. Patient Selection

The characteristics of patients and lesions are summarized in Table 1. Mean ages of patients with pituitary adenoma and Rathke cleft cyst were 48.57 years and 45.19 years, respectively. Most lesions were located in the intrasellar and suprasellar region regardless of the tumor types. The average size of pituitary adenoma was 25.72 mm, and Rathke cleft cyst was 21.14 mm. Among the 83 patients with pituitary adenoma, 49 were clinically nonfunctioning adenoma, 16 were acromegaly, 9 were Cushing disease, 5 were gonadotropin-secreting adenoma, and 4 were prolactinoma.

### 3.2. Analysis of MR Image Features

**Figure 1:**
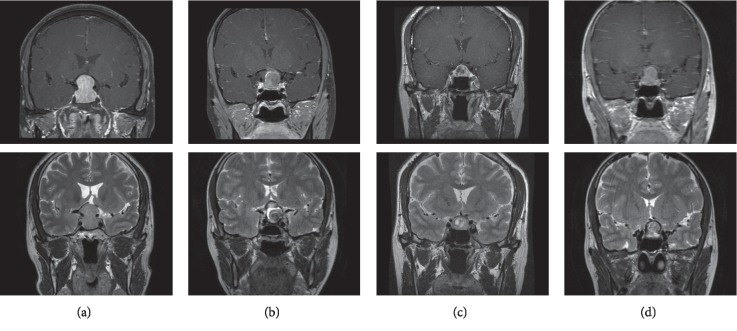
Examples of pituitary adenoma and Rathke cleft cyst on the contrast-enhanced image and T2WI. (a) Typical pituitary adenoma. (b) Typical Rathke cleft cyst. (c) Atypical pituitary adenoma (cystic pituitary adenoma after necrosis). (d) Atypical Rathke cleft cyst.

Like texture features, the evaluation of MR image features was basically based on the contrast-enhanced image and T2WI (Figure 1). All participants in the training group had the preoperative contrast-enhanced image, and T2WI was available in 90 patients. Among the five MR image features we evaluated, signal intensity on the contrast-enhanced image (*p* < 0.001) and signal intensity on T2WI (*p*=0.028) were observed significantly different between pituitary adenoma and Rathke cleft cyst. Benjamini–Hochberg correction adjusted the level of significance *p*^*∗*^=0.01, making signal intensity on the contrast-enhanced image as the only significant MR image feature. Hyperintense on the contrast-enhanced image was more likely to represent in pituitary adenoma, while hypointense or isointense was more likely to represent in Rathke cleft cyst. The other image features, including flow voids, heterogeneity on the contrast-enhanced image, and heterogeneity on T2WI, did not differ significantly between these two lesions (Table 2).

### 3.3. Analysis of Texture Features

**Figure 2:**
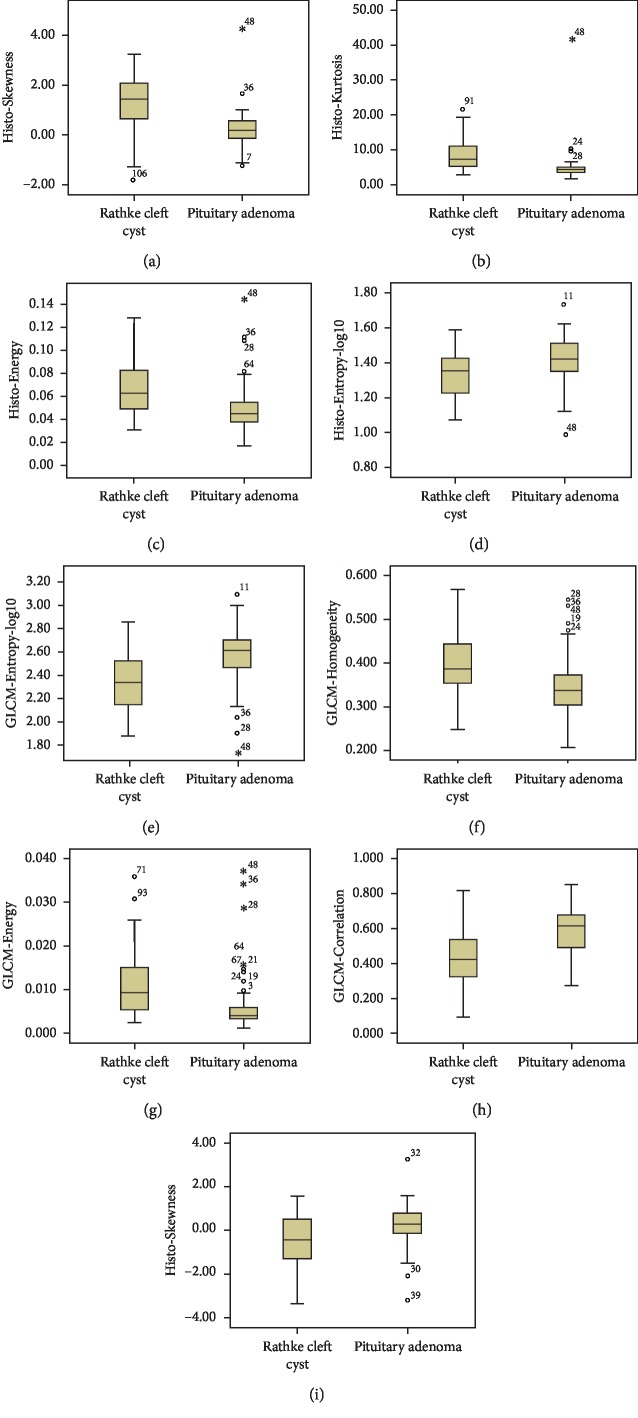
Boxplot of nine significantly different texture features in discriminating pituitary adenoma from Rathke cleft cyst. (a)∼(h) Texture features from the contrast-enhanced image; (i) texture features from T2WI.

**Figure 3:**
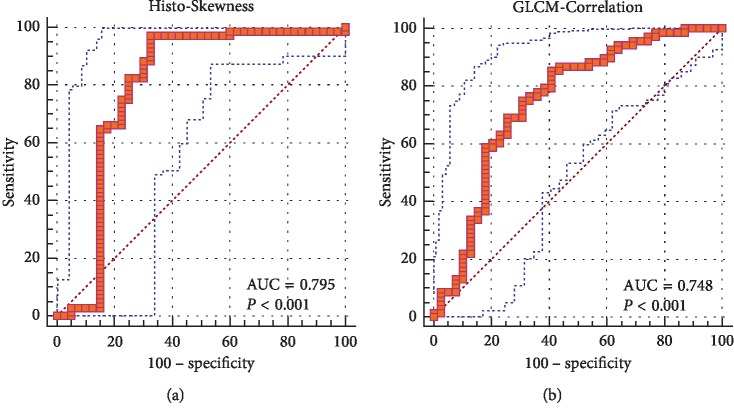
ROC curves of two independent predictors from the contrast-enhanced image in discrimination between pituitary adenoma and Rathke cleft cyst.

A total of nine features differed significantly between pituitary adenoma and Rathke cleft cyst according to the Mann–Whitney *U* test in training group (Table 3). On the contrast-enhanced image, compared with Rathke cleft cyst, pituitary adenoma was associated with lower Histo-Energy (<0.001), higher Histo-Entropy (0.002), lower Histo-Kurtosis (<0.001), lower Histo-Skewness (<0.001), higher GLCM-Correlation (<0.001), lower GLCM-Energy (<0.001), higher GLCM-Entropy (<0.001), and lower GLCM-Homogeneity (0.001) (Figures 2(a)∼2(h)). On T2WI, pituitary adenoma demonstrated higher Histo-Skewness (0.004) than Rathke cleft cyst (Figure 2(i)). The above nine features were also significant after adjusting the level of significance *p*^*∗*^=0.005. Since Histo-Energy, Histo-Entropy, GLCM-Contrast, GLCM-Dissimilarity, GLCM-Energy, GLCM-Entropy, and GLCM-Homogeneity on the contrast-enhanced image were all collinear correlated, we performed binary logistic regression analysis on GLCM-Dissimilarity from the contrast-enhanced image and the other three variables from the contrast-enhanced image (Histo-Kurtosis, Histo-Skewness, and GLCM-Correlation). The results of binary logistic regression analyses on the above four standardized variables suggested that Histo-Skewness and GLCM-Correlation from the contrast-enhanced image could be taken as independent predictors (Table 4). Then ROC analysis was performed on the two independent predictors to assess their discriminative ability in the training group. Histo-Skewness from the contrast-enhanced image whose AUC value was 0.80 had the highest discriminatory power and implied the practical value. On the other hand, the AUC value of GLCM-Correlation was 0.75 (Figure 3). The AUC value, standard error, 95% CI, cutoff point, sensitivity, and specificity of each feature are listed in Table 5. In the validation group, Histo-Skewness from the contrast-enhanced image represented better discriminative performance with accuracy of 0.80, sensitivity of 0.93, and specificity of 0.60. The accuracy, sensitivity, and specificity of GLCM-Correlation from the contrast-enhanced image were 0.72, 0.80, and 0.60, respectively, suggesting that it also had feasible ability in differentiation.

### 3.4. Relationship between MR Image Features and Texture Features

**Figure 4:**
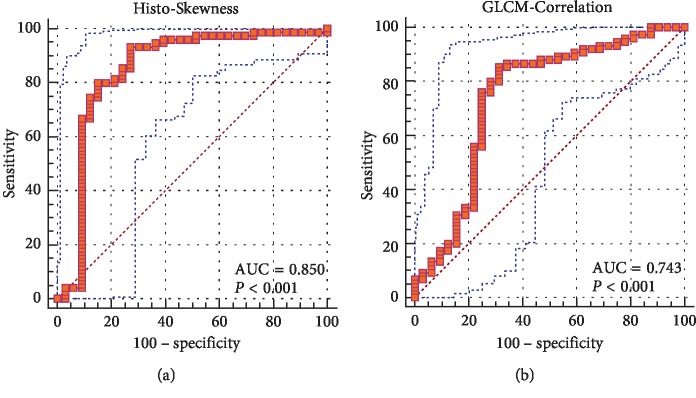
Relationship between MR image feature (signal intensity on the contrast-enhanced image) and texture features (Histo-Skewness and GLCM-Correlation from the contrast-enhanced image).

We performed analysis on significantly different MR image feature (signal intensity of the contrast-enhanced image) and the two texture features which could be taken as independent predictors (Histo-Skewness and GLCM-Correlation from the contrast-enhanced image) to explore their relationship. According to the Mann–Whitney *U* test, significant differences were observed in both Histo-Skewness and GLCM-Correlation on the contrast-enhanced image (Supplementary [Supplementary-material supplementary-material-1]). Subsequent ROC analysis demonstrated that the AUC values of Histo-Skewness and GLCM-Correlation on the contrast-enhanced image were 0.85 and 0.74, respectively, suggesting that these two texture features were related to signal intensity on the contrast-enhanced image (Figure 4). Detailed results, including *p* value, AUC value, standard error, 95% CI, optimal cutoff point, sensitivity, and specificity are summarized in Table 6.

## 4. Discussion

In the present study, we combined magnetic resonance scan and texture analysis to differentiate pituitary adenoma from Rathke cleft cyst. Besides, we also investigated the relationship between MR image features and texture features. To our best knowledge, texture analysis had never been performed between these two types of lesions, making our study to be the first one to establish the differences between the two lesions macroscopically and microcosmically.

Magnetic resonance scan had long been recognized as the most useful technique to distinguish pituitary adenoma from Rathke cleft cyst. Thus, our study first evaluated the ability of qualitative MR image features to discriminate these two lesions. We found that signal intensity on the contrast-enhanced image showed significant ability in discrimination. To be more specific, pituitary adenoma was more likely to show hyperintense on the contrast-enhanced image, while Rathke cleft cyst rarely showed enhancement, which was consistent with the previous studies [7, 20, 21]. However, heterogeneity on the contrast-enhanced image did not differ significantly between two lesions. A possible explanation for this might be that the heterogeneity of Rathke cleft cyst could be volatile arising from the variation of protein concentration within the cystic fluid. On the other hand, signal intensity and heterogeneity on T2WI did not show significant difference between two lesions, which might also be arisen from the variable protein concentration within the cystic fluid of Rathke cleft cyst. Therefore, signal intensity on the contrast-enhanced image could be taken as the most valuable MR image feature in discrimination. However, the descriptions of the above MR image features largely depended on the experience of radiologists and were relatively subjective, and the volatile signal in MR images of Rathke cleft cyst made the situation confusing even for experienced senior radiologists [8].

Texture analysis was a novel radiological tool which could extract useful features from images and obtain quantitative results by the software. Many studies had demonstrated that texture analysis from MRI had promising and practical ability in detection, differentiation, and prognosis prediction of various types of tumors [22–26]. As for brain tumors, it had been performed on glioma, medulloblastoma, meningioma, and primary central nervous system lymphoma [27–29]. However, it had never been conducted in pituitary adenoma or Rathke cleft cyst, and the previous studies which were usually limited by the number of patients required statistical supplement [10, 13, 30, 31]. Our study was the first study to perform this technique in the discrimination between pituitary adenoma and Rathke cleft cyst. More importantly, our study had a relatively larger sample size than earlier studies and obtained two independent predictors in differentiation through binary logistic regression analysis: Histo-Skewness and GLCM-Correlation from the contrast-enhanced image. Histo-Skewness, which measured the asymmetry of the grey-level distribution in the histogram, was found higher in Rathke cleft cyst; GLCM-Correlation, which reflected linear dependency of grey-levels in GLCM, was found higher in pituitary adenoma [32]. Moreover, Histo-Skewness from the contrast-enhanced image whose AUC value was 0.80 had the highest discriminatory power and could make practical value. Thus, texture analysis which provided nonvisual information of the image could be a helpful tool to assist in clinical diagnosis.

The relationship between two types of features was analyzed, considering both features had promising ability in discrimination. Many studies had implied that texture features might be associated with histopathological characteristics of the lesion, such as cellular density, heterogeneity, level of vascularization, and microstructural changes [10, 27, 30, 32]. Meanwhile, the above factors could contribute to MR features of the lesion to some extent. Generally, pituitary adenoma with relatively high cellular density typically showed solid characteristics and homogeneous enhancement, while Rathke cleft cyst presented with a cystic lesion and lacked enhancing component, resulting from no vascularization inside its cystic fluid. Therefore, we conducted analysis to investigate the relationship between MR image features and texture features. Encouragingly, subsequent analysis proved our hypothesis, showing that both Histo-Skewness and GLCM-Correlation were related to signal intensity on the contrast-enhanced image. This result proved the connection between MR image features and texture features and indicated that our study was objective and convincing, as we linked the two sets of features together.

Our study had several limitations. First, it was a retrospective, single-center study and patients with preoperative MR scan were only included, and the selection bias was enrolled inevitably. Second, we only analyzed texture features extracted from Histogram-based matrix and GLCM, which were the most common reported matrices in the previous studies [24, 29, 30, 33]. Third, we were unable to evaluate the value of texture analysis on conventional T1WI. Forth, in this study, the two types of lesions which were all screened from the neurosurgery department usually had arisen mass effect, so they were relatively large in size. Furthermore, four patients with prolactinoma received bromocriptine before undergoing surgery in our neurosurgery department, which might have an uncertain effect on MR features and texture features. However, we were unable to illustrate its specific influence in the present study due to the scarce information of patients and requirement of ethics committee that only statistics in our neurosurgery department were available for analysis, requiring future study to explore the influence of preoperatory treatment. Future studies on multiple centers and other matrices were needed to validate our results and to investigate the value of texture analysis in discrimination prospectively.

## 5. Conclusion

In conclusion, signal intensity on the contrast-enhanced image was the most significant MR image feature in discrimination of pituitary adenoma from Rathke cleft cyst. Moreover, the texture analysis on the contrast-enhanced image, which could reflect the MR image features, had the potential to be served as radiomic parameters in differentiation.

## Figures and Tables

**Table 1:** Characteristics of patients and lesions.

Characteristics	Pituitary adenoma (*n* = 83)	Rathke cleft cyst (*n* = 50)
Age (year), mean (range)	48.57 (18∼73)	45.19 (21∼68)
Gender		
Male	39	23
Female	44	27
Tumor size (mm)		
Mean ± SD	25.72 ± 9.01	21.14 ± 7.91
Range	7∼50.5	8∼38.3
Tumor location		
Intrasellar	26	15
Intrasellar and suprasellar	57	35
Days between MRI and surgery (day), mean (range)	13.87 (1∼101)	15.02 (1∼68)

MRI, magnetic resonance imaging; SD, standard deviation.

**Table 2:** Differences of qualitative MR image features between pituitary adenoma and Rathke cleft cyst in the training group.

Qualitative MR image features	Pituitary adenoma, *N* (%) (*n* = 68)	Rathke cleft cyst, *N* (%) (*n* = 40)	*p* value (<0.01)^*∗*^
Flow voids			
Presence	3 (4)	2 (5)	0.866
Absence	65 (96)	38 (95)	(Fisher's exact tests)
Signal intensity on the contrast-enhanced images			
Hypointense or isointense	3 (4)	30 (75)	**<0.001**
Hyperintense	65 (96)	10 (25)	(chi-square tests)
Heterogeneity on the contrast-enhanced images			
Homogenous	34 (50)	25 (62.5)	0.208
Heterogeneous	34 (50)	15 (37.5)	(chi-square tests)
Signal intensity on T2WI			
Hypointense or isointense	35 (64)	14 (40)	0.028
Hyperintense	20 (36)	21 (60)	(chi-square tests)
Heterogeneity on T2WI			
Homogenous	27 (49)	20 (57)	0.456
Heterogeneous	28 (51)	15 (43)	(chi-square tests)

MR, magnetic resonance; T2WI, T2-weighted image. ^*∗*^Correction adjusted level of significance *p*=0.01.

**Table 3:** Differences of texture features between pituitary adenoma and Rathke cleft cyst on the contrast-enhanced image and T2WI based on the Mann–Whitney *U* test.

Texture feature, mean (range)	Contrast-enhanced image			T2WI		
	Pituitary adenoma	Rathke cleft cyst	*p*	Pituitary adenoma	Rathke cleft cyst	*p*
Histo						
Energy	0.05 (0.02∼0.14)	0.07 (0.03∼0.13)	**<0.001**	0.05 (0.03∼0.13)	0.05 (0.02∼0.14)	0.263
Entropy	1.42 (1.00∼1.75)	1.33 (1.07∼1.59)	**0.002**	1.40 (1.11∼1.62)	1.45 (1.07∼1.68)	0.078
Kurtosis	4.93 (1.70∼41.27)	8.82 (2.91∼21.31)	**<0.001**	6.07 (1.59∼28.98)	5.40 (1.51∼20.23)	0.599
Skewness	0.25 (−1.19∼4.67)	1.25 (−1.72∼3.23)	**<0.001**	0.27 (−2.87∼3.34)	−0.43 (−3.37∼1.56)	**0.004**

GLCM						
Correlation	0.58 (0.27∼0.85)	0.44 (0.09∼0.82)	**<0.001**	0.52 (0.11∼0.89)	0.45 (−0.34∼0.86)	0.173
Contrast	41.01 (6.64∼161.70)	43.19 (4.54∼154.60)	0.600	49.76 (11.81∼146.29)	84.69 (9.40∼717.59)	0.028
Dissimilarity	4.31 (1.66∼9.10)	3.98 (1.39∼8.74)	0.310	4.60 (2.25∼8.63)	5.64 (1.852∼22.209)	0.108
Energy	0.01 (0.00∼0.03)	0.01 (0.00∼0.04)	**<0.001**	0.01 (0.00∼0.03)	0.01 (0.00∼0.03)	0.566
Entropy	2.60 (1.76∼3.08)	2.34 (1.87∼2.85)	**<0.001**	2.60 (1.91∼3.00)	2.52 (1.85∼2.98)	0.579
Homogeneity	0.30 (0.21∼0.54)	0.40 (0.25∼0.57)	**0.001**	0.30 (0.22∼0.51)	0.34 (0.16∼0.53)	0.984

T2WI, T2-weighted image; Histo, histogram-based matrix; GLCM, grey-level co-occurrence matrix.

**Table 4:** Binary logistic regression analysis on texture features.

Texture features			OR	95% CI	*p* value
Contrast-enhanced image	Histo	Skewness	0.352	0.166∼0.748	**0.007**
		Kurtosis	1.395	0.496∼3.922	0.528
	GLCM	Correlation	2.734	1.391∼5.372	**0.004**
		Dissimilarity	1.643	0.79∼3.415	0.183

OR, odds ratio; CI, confidence interval; Histo, histogram-based matrix; GLCM, grey-level co-occurrence matrix.

**Table 5:** The diagnostic ability of independent predictors in discriminating pituitary adenoma from Rathke cleft cyst in the training group.

Texture features	AUC	Standard error	95% CI	Cutoff point	Sensitivity	Specificity
Contrast-enhanced image						
Histo-Skewness	0.80	0.056	0.707∼0.867	1.005	97.06	67.50
GLCM-Correlation	0.75	0.053	0.655∼0.827	0.450	85.29	58.97

Histo, histogram-based matrix; GLCM, grey-level co-occurrence matrix; AUC, area under the curve; CI, confidence interval.

**Table 6:** The results of the receiver operating characteristics curve for relationship between MR image feature (signal intensity on the contrast-enhanced image) and texture features from the contrast-enhanced image.

Texture features	*p* value	AUC	Standard error	95% CI	Cutoff point	Sensitivity	Specificity
Contrast-enhanced image							
Histo-Skewness	**<0.0001**	0.85	0.050	0.768∼0.911	1.005	93.33	72.73
GLCM-Correlation	**<0.0001**	0.74	0.059	0.649∼0.822	0.450	85.33	68.75

Histo, histogram-based matrix; GLCM, grey-level co-occurrence matrix; AUC, area under the curve; CI, confidence interval.

## Data Availability

The data used to support the findings of this study are available from the corresponding author upon request.

## Supplementary Materials

Supplementary Materials Supplementary Table 1: the results of the Mann–Whitney *U* test for investigating the association between MR image features (signal intensity on the contrast-enhanced image) and texture features from the contrast-enhanced image.
