# Supplementary material for: Differentiation of Pituitary Adenoma from Rathke Cleft Cyst: Combining MR Image Features with Texture Features
Source: Contrast Media Mol Imaging. 2019 Oct 28;2019:6584636. doi: 10.1155/2019/6584636 (PMC6854938; doi:10.1155/2019/6584636)
Supplement: Supplementary Materials — Supplementary Table 1: the results of the Mann–Whitney U test for investigating the association between MR image features (signal intensity on the contrast-enhanced image) and texture features from the contrast-enhanced image. [file 6584636.f1.docx]

**Supplement Table 1.** The results of Mann-Whitney U test for investigating the association between MR image feature (signal intensity on contrast-enhanced image) and texture features from contrast-enhanced image.

| Texture features | P-value |
| --- | --- |
| contrast-enhanced image  Histo-Skewness  GLCM-Correlation |  |
|  | **<0.001** |
|  | **<0.001** |

Abbreviations: Histo, Histogram based matrix; GLCM, Grey-level co-occurrence matrix.
